# A bell that never rang: Accompanying a child through an endless class

**DOI:** 10.1017/S1478951526103101

**Published:** 2026-07-13

**Authors:** Hsuan-Ting Ho, Ming-Jen Chan, Tang-Her Jaing

**Affiliations:** 1 Department of Medicine, Chang Gung Medical College, Taiwan; 2 Nephrology and Kidney Research Center, Chang Gung Memorial Hospital Linkou Main Branch, Taoyuan, Taiwan; 3 Department of Pediatrics, Chang Gung Memorial Hospital Linkou Branch, Taoyuan, Taiwan

September 8, 2025, was my first day on rotation in the pediatric hematology-oncology ward. That same day, an 8-year-old boy was admitted early – spiking fever before his scheduled chemotherapy – and became my primary care patient. For me, it was the beginning of a new rotation; for him, it was simply one more time pushing open the hospital doors, as he had done countless times before in his long fight against disease.

His medical chart read like a chronicle of relentless battle: retinoblastoma discovered when he was just 8 months old, barely able to sit up. That chapter finally closed in 2019. In 2023, secondary acute myeloid leukemia (AML) was diagnosed, leading to a bone marrow transplant in September 2024. After 6 months of relative stability, July 2025 brought the news no one wanted: AML relapsed.

When I first met him in the ward, all those heavy diagnoses seemed to vanish. He was curled up small inside his large hospital bed – a quiet, delicate boy. One eye fixed slightly toward the midline, yet his eyes were still wide and dark, his skin a pale, luminous white. He looked like any boy who should have been racing across a schoolyard. He was just living in a hospital instead.

It was not until the day of his lumbar puncture that I truly saw his body. Though he measured 125 cm tall, he weighed only 16 kg – his limbs so thin they made his head look disproportionately large. Since this admission, worsening bone pain had slowly stolen his ability to walk.

In that moment, I understood: this disease had truly eaten away at a child’s body – eroding, piece by piece, the shape of the healthy boy he should have become (Sourkes 1995).

The nursing practitioners adored him. Once, before a procedure, he pressed his lips together – the silent, dignified grief of a child trying not to cry, tears pooling but not falling. When the nurse returned to the station and found the candy box empty of his favorite brand, she immediately sent a colleague to buy the specific kind he loved. In a pediatric ward, care is not only medication. It is also the tenderness so fine it nearly goes unnoticed – never charted, never documented.

And he was remarkably intelligent. Every morning on rounds, he asked: “What is my white blood cell count today?” “How are my platelets?” “Do I need a transfusion?” When the numbers improved, he would light up: “Oh, that’s better than yesterday!” He had a command of his own medical situation that exceeded his years – he understood what each day’s orders signified.

Yet behind that precocious knowledge, he remained entirely a child. He would solemnly introduce me to his collection of French fry plushies, each one with its own name. His favorite, Little Do, had come with him for this admission. He held it while he talked, reassured it, “it doesn’t hurt, it doesn’t hurt,” and even shared his food with it. His side of the bed was always filled with the soft, continuous murmur of his voice.

He would often ask us, with a mixture of hope and careful testing:

Can I be dismissed today?

Children in the pediatric hematology-oncology ward do not say “discharge.” They call the intervals between chemotherapy cycles – the times they can leave the hospital – “being let out of class.” The hospital is a class without a bell. Medication, vomiting, blood drawing, waiting: one period following another, with no true ending.

## The longest class

The moment I remember most vividly was a morning round. He looked up at the attending physician – the doctor who had cared for him for years – and said:

I want to be a doctor like you when I grow up.

The attending physician smiled and replied, “Then you have to be good and listen to us.”

Without missing a beat, he answered:

I am good. So you have to cure me.

When those words landed, the room seemed to go briefly silent. My eyes burned. He simply, sincerely believed: I have tried hard, I have been patient, I have been brave – so can you make me well? The most devastating thing was that everyone in the room knew medicine cannot always answer that kind of faith.

## Time’s unequal weight

My rotation in pediatric hematology-oncology ended on September 12^th^. In their language, I graduated. In the months that followed, whenever I thought of him, I would go back and check his chart – I had memorized the attending physician’s code. On December 12^th^, as I was preparing for winter vacation, I checked again – and discovered he had been confined to the ward for nearly 90 days. For me, rotations ended and vacations began; time moved forward. For him, time seemed pinned inside the hospital, cycling through infections, chemotherapy, and waiting.

He had asked so many times when class would let out. Class never came.

After that, I spent 2 weeks of a nearly carefree holiday, until I returned to the clinic in February. A narrative medicine assignment (Laskow 2019) brought him back to my mind, and I searched for the attending physician’s code – his name was no longer on the patient list. Relieved for a moment, I searched by his medical record number instead and found that his story had ended on December 22^nd^.

While I had briefly forgotten the hospital, forgotten the ward, forgotten all that suffering and struggle – he had not lived to see the new year.

I read backward, entry by entry. He had managed a discharge in mid-December, only to be brought back to the pediatric emergency room in the early hours of December 22^nd^ by his father, cold and struggling to breathe. The chart noted hospice care; the medical orders confirmed his family had signed a DNR. After he was transferred upstairs at seven o’clock, the nurses recorded his vital signs nearly every half hour.

At 10:00, he suddenly wanted bubble tea. His mother ran to buy it. His beloved nursing practitioner stayed at his bedside.

At 10:30, when his mother returned, he drank 2 sips and looked happy – then grew too breathless to sit up and lay back down.

At 10:50, his heart rate and blood pressure were undetectable.

He passed away quietly – beside his dearest mother, his nurses who cherished him, and the attending physician who had walked with him for 7 years. His life was fixed at 8 years and 7 months old. When I read that record, I felt both grief and something like consolation: he had finally been let out of class. Though no class had ever asked so much of anyone.

## What care can hold

What grieved me most was not only his death, but the cruel asymmetry of time between us. My happiest, most carefree winter holiday was the ending of his life.

I used to wonder whether palliative care was too powerless. It cannot cure the disease. It cannot spare a family from breaking. What can it actually leave for a child who spent nearly his entire life fighting illness (Wiencek 2024)?

Perhaps palliative care is not about making death acceptable. Perhaps it is about preserving, within an unavoidable departure, the small things a person holds dear (Engel 2023).

For this child, those things were small. Someone who remembered he was afraid of pain. A particular brand of candy. People willing to learn the names of his French fry plushies. And on that final day, the 2 sips of bubble tea his mother ran to bring him. Measured against cure, none of these changed the outcome. Measured against care, they meant everything – because they meant he was not alone at the end.

Looking back, I understand now that “witnessing” in narrative medicine is not only about seeing the patient (Charon 2001; Bingley 2008). It is also about seeing the family. I saw his gentle mother – who, in the midst of a situation that had utterly exhausted her, still made space for me, an uncertain medical student, to ask my slow questions and slowly learn. I saw how she stood at the edge between hope and despair and continued, with great effort, to love her child. As I write this, I find myself returning again and again to his face: the seriousness when he introduced his plushies, the moment he said “I am good, so you have to cure me” – with such unquestioning trust in the adults around him. Every memory carries a weight that is both helpless and tender.

“To cure sometimes, to treat often, to comfort always” – a phrase so familiar it has almost worn smooth. I am still not certain that palliative care fully answered what he was asking for. What he truly wanted was the chance to grow up. But at the very least, through that long and difficult class, a group of people stayed beside him – and helped him complete that slow, impossible, lifelong dismissal.
